# Citation report: the nightmares and dreams of the editor: we grew 30%

**DOI:** 10.5935/0004-2749.202100112

**Published:** 2021

**Authors:** Eduardo M. Rocha, Rodrigo Pessoa Cavalcanti Lira, Silvana Artioli Schellini, Rosalia Antunes-Foschini

**Affiliations:** 1 Faculdade de Medicina de Ribeirão Preto, Universidade de São Paulo, Ribeirão Preto, SP, Brazil; 2 Universidade Federal de Pernambuco, Recife, PE, Brazil; 3 Faculdade de Medicina, Universidade Estadual de São Paulo “Júlio de Mesquita Filho”, Botucatu, SDP, Brazil; 4 Hospital das Clínicas, Faculdade de Medicina de Ribeirão Preto, Universidade de São Paulo, Ribeirão Preto, SP, Brazil

The publication of the Citation Report and its most relevant number, the Journal Impact Factor (JIF), by Journal Citation Reports (JCR) in the Web of Science, which is sponsored by Clarivate Analytics, is a medium-term evaluation of scientific journals. The JIF is an index calculated by dividing the number of citations of the articles published in the JCR year “y” by the total number of citable articles published in the two previous years (“y-1” and “y-2”) in a given journal^(1)^.

ABO has grown 29.2% since the last report, from 0.617 to 0.872. It is still below 1.0, considering it has only been followed since its inclusion in the Web of Science in 2010, except for 2017, when a special report on Zika virus resulted in hundreds of citations in the months following its publication.

This growth is a result of honest, unpaid, and unselfish work of reviewers and members of the editorial board. It is a conquest for a Brazilian journal that has been published in Portuguese for almost 70 years and only in print in the majority of its 85 years in publication^(2)^. ABO editors are aware that the JIF, like the p-value, is a controversial element in academic life, and its utility requires caution^(3,4)^. Hence, we should not summarize the assessments of individual researchers and their results using metrics for more prominent journals.

We want more in the future. We are not just dreaming, and the reasons are: we have a qualified editorial board composed of experienced professors, ophthalmologists, and researchers who are recognized for their academic merits around world; we are receiving manuscripts and letters from all continents; and the journal is open access, free for authors, and equipped with the most advanced tools and rules of scientific publication.

In 2010, when it entered the Web of Science and JCR, ABO was among the 56 journals in the ophthalmological field. In the last JCR in 2020, the list of journals in ophthalmology was 62; in 2021, the list has increased to 89. We would like to congratulate the editorial board on the excellent performance of the journals listed in JCR and welcome the new journals. We know from experience that hard work and commitment are behind the numbers presented in this report. Comparative analysis allows for a healthy completion for quality in editorial work.

We would also like to express our gratitude to our rea ders, authors, reviewers, editors, and our sponsor, The Brazilian Council of Ophthalmology. They are the basis for the future of ABO. Editorship is an everyday team commitment, and the efforts of this team will be the antidote against the insomnia and nightmare of being downgraded. In this way, we trust that ABO will continue to be among the best scientific journals in the world.
